# Mental health of women and children experiencing family violence in conflict settings: a mixed methods systematic review

**DOI:** 10.1186/s13031-021-00410-4

**Published:** 2021-10-15

**Authors:** Delan Devakumar, Alexis Palfreyman, Amaran Uthayakumar-Cumarasamy, Nazifa Ullah, Chavini Ranasinghe, Nicole Minckas, Abhijit Nadkarni, Sian Oram, David Osrin, Jenevieve Mannell

**Affiliations:** 1 Institute for Global Health, University College London, London, WC1N 1EH UK; 2 Hull University Teaching Hospitals NHS Trust, Hull, UK; 3 UCL Medical School, 74 Huntley Street, London, UK; 4 London School of Hygiene and Tropical Medicine, London, UK; 5 Sangath, Porvorim, India; 6 King’s College London, London, UK

## Abstract

**Background:**

Armed conflict has significant impacts on individuals and families living in conflict-affected settings globally. Scholars working to prevent violence within families have hypothesised that experiencing armed conflict leads to an increase in family violence and mental health problems. In this review, we assessed the prevalence of family violence in conflict settings, its association with the mental health of survivors, moderating factors, and the importance of gender relations.

**Methods:**

Following PRISMA guidelines, we systematically reviewed quantitative and qualitative studies that assessed the prevalence of family violence and the association between family violence and mental health problems, within conflict settings (PROSPERO reference CRD42018114443).

**Results:**

We identified 2605 records, from which 174 full text articles were screened. Twenty-nine studies that reported family violence during or up to 10 years after conflict were eligible for inclusion. Twenty one studies were quantitative, measuring prevalence and association between family violence and mental health problems. The studies were generally of high quality and all reported high prevalence of violence. The prevalence of violence against women was mostly in the range of 30–40%, the highest reported prevalence of physical abuse being 78.9% in Bosnia and Herzegovina. For violence against children, over three-quarters had ever experienced violence, the highest prevalence being 95.6% in Sri Lanka. Associations were found with a number of mental health problems, particularly post-traumatic stress disorder. The risk varied in different locations. Eight qualitative studies showed how men’s experience of conflict, including financial stresses, contributes to their perpetration of family violence.

**Conclusions:**

Family violence was common in conflict settings and was associated with mental health outcomes, but the studies were too heterogenous to determine whether prevalence or risk was greater than in non-conflict settings. The review highlights an urgent need for more robust data on perpetrators, forms of family violence, and mental health outcomes in conflict-affected settings in order to help understand the magnitude of the problem and identify potential solutions to address it.

**Supplementary Information:**

The online version contains supplementary material available at 10.1186/s13031-021-00410-4.

## Introduction

Rates of family violence and mental ill health are hypothesised to be higher in areas of armed conflict than in non-conflict-affected settings [1], but the extent of the risks and their associations have not been systematically assessed.

Family violence, including violence against women (VAW) from intimate partners or other household members and violence against children, is prevalent across the world and has adverse implications for physical and mental health [2]. The WHO multi-country study on women’s health and domestic VAW, showed that lifetime prevalence of physical and sexual abuse by an intimate partner ranges from 15 to 71% [3]. Rates of child abuse vary by type of abuse, definition and location. ‘Base-case estimates’ from 96 countries suggested at least 50% prevalence of exposure to violence in the last year, equating to approximately 500 million children aged 2–17 years [4]. A systematic review of 55 studies estimated the prevalence of sexual violence to be 8–31% in girls and 3–17% in boys [5]. Sexual violence in particular is associated with a long-term increased risk of mental and physical illness [6].

Reports from conflict settings indicate increased rates of VAW and physical and sexual abuse of children by both men and women [7–9]. For example, in a study of child soldiers from Sierra Leone, 44% reported having been raped [10]. There are a number of possible reasons for this. The social and economic conditions that lead to conflict at a societal level may also result in increases in family violence. Catani et al. propose that the ‘cycle of violence’ model, in which violence is transmitted intergenerationally within a family [11], can also apply to conflict situations in which external violence leads to violence within the family [12]. The mechanisms by which this may occur are multiple. Conflict may normalise violence in a society, increase substance abuse, and affect education, income, family composition, and gender attitudes [13–15]**.**

In addition to violence, mental disorders are common in conflict, due to both the conflict itself and the family violence. Women and children living in conflict-affected settings are at increased risk of developing depressive, anxiety, and psychotic disorders [16, 17]. The prevalence of mental disorders in conflict-affected populations is substantially higher than in the average population: 15.4% for post-traumatic stress disorder (PTSD) and 17.3% for depression, versus 7.6% for any anxiety disorder including PTSD and 5.3% for any mood disorder including depressive disorders [18, 19].

There remains a gap in knowledge on the prevalence of family violence in conflict-affected areas and its mental health consequences. Greater understanding is needed of why and how family violence may result in an increase in mental disorders in conflict-exposed populations. This review was designed to explicitly investigate links between family violence occurring at an individual level (within families), and broader forms of violent armed conflict occurring within communities. We included both quantitative and qualitative research to both enumerate the problem and explore the reasons why armed conflict is associated with mental ill-health, expanding our conceptual understanding of the underlying causes. Our review had four objectives. First, to investigate the prevalence of family violence, including violence against women and children, in conflict-affected areas. Second, to describe the risk of mental disorder among women and children who had experienced or witnessed family violence in conflict-affected areas. Third, to examine how the association of family violence with mental health in women and children is moderated by the type of conflict, the gender of the perpetrator, and the type of violence. Finally, to understand the ways in which gender relations, as a well-recognised risk factor for violence against women and children [20], influence the mental health of women and children who experience or witness family violence.

## Methods

### Design

We conducted a mixed-methods systematic review. The protocol was registered prospectively with PROSPERO (CRD42018114443) and reporting follows PRISMA guidelines [21].

### Search strategy

We searched the following databases: EMBASE, International Bibliography of Social Sciences, MEDLINE (PubMed), PsycINFO, Scopus, SciELO, Social Policy and Practice, Sociology database, Global Index Medicus, Online Library of Dignity, Web of Science, Cochrane Library, CINAHL Plus, and regional databases (LILACS, African Journals online, Latin America & Iberia Database (ProQuest), Middle East & Africa Database (ProQuest)). Studies were imported into Endnote and duplicates removed. The search was restricted to articles in English, Arabic, Spanish, Portuguese, and French, with no date restrictions. Database searches were complemented by reference list screening and citation tracking of included materials in Web of Science and Google Scholar.

### Search terms

The search strategy (Additional file 1: Appendix) was designed with the support of a librarian and included terms related to (1) women and children, (2) family violence, (3) mental disorders, and (4) conflict-affected areas. Terms describing conflict-affected areas were compiled using a list of countries and territories in which armed conflicts were recorded before 2018, using the Uppsala Conflict Data of the International Peace Research Institute and the Heidelberg Institute for International Conflict Research. We used the Uppsala Institute interstate and intrastate definitions of conflict: “An armed conflict is a contested incompatibility that concerns government and/or territory where the use of armed force between two parties, of which at least one is the government of a state, results in at least 25 battle-related deaths in one calendar year”; and “A conflict between a government and a non-governmental party, with no interference from other countries” [22].

### Inclusion criteria

*Population and setting:* Our review included adult women (aged 18 years or older), and male or female children (aged under 18 years). If the study sample included men aged 18 or older, reports were only included if 90% were under 18. We did not include facility-based studies in the assessment of prevalence due to the risk of selection bias, but chose to include school studies to reflect the universal right to education. We included studies up to 10 years post-conflict to capture the ongoing effects of war. *Type of study:* We included primary research using qualitative (e.g. individual interviews, focus group interviews) or quantitative (e.g. cohort, case–control, cross-sectional studies, or baseline data from experimental or quasi-experimental studies) research designs. Where quantitative studies were reported in more than one publication, the one with the largest sample was selected for inclusion. *Exposures:* We included family violence against women or children. The definition of family violence, adapted from the United Nations definition of violence against women [23] was the intentional use of physical force or power by an intimate partner, ex-partner or parent that results in physical, sexual or mental harm or suffering to women and/or children. This includes threats of violence, sexual coercion, psychological abuse and arbitrary deprivation of liberty. Family violence is an overarching term that includes intimate partner violence and child abuse, perpetuated by family members. Studies of children who witness violence against women in the home in addition to experiencing direct violence themselves were included. The comparator group for quantitative studies was no exposure to family violence. *Outcomes:* Our primary outcome was mental disorder, defined in accordance with the International Classification of Diseases and Related Health Problems (ICD-11) or Diagnostic and Statistical Manual of Mental Disorders, 5th Edition (DSM-V) criteria. Articles were eligible if mental disorder was assessed using either a validated diagnostic or screening instrument, diagnosed by a clinician, or self-reported by participants. The secondary outcomes included were mental and social wellbeing of women experiencing family violence and children experiencing or witnessing family violence.

### Exclusion criteria

*Population and setting:* We excluded soldiers and war veterans, refugees and asylum seekers who had moved away from the conflict setting. Soldiers and war veterans were considered to be too different a population who had experienced combat directly. *Type of study:* We excluded opinion pieces and editorials, dissertations or theses, policy papers, reviews, general reports that did not introduce new evidence from a specific study, and conference abstracts. *Exposures:* Other forms of violence against women and children were beyond the scope of the review; for example, peer bullying, violence by perpetrators other than intimate partners, ex-partners, or family members, female genital mutilation or cutting, or child labour or marriage.

### Screening

Titles and abstracts were screened independently by two reviewers (CR, NU) from 2nd June 2019. Full texts were screened from 31st July 2019. Data extraction began on 1st September 2019 and was completed by January 2020. Disagreement was resolved by discussion and no papers were discussed with a third reviewer. Each full text was then independently reviewed by two authors (two of AP, AU-C, CR, DD, NU, JM). Disagreement was resolved by discussion. One author was contacted for missing data, but did not respond.

### Quality assessment

Two reviewers assessed each article (qualitative articles by AP and JM, with disagreements decided by DD; quantitative articles by A-UC and DD) for quality using the Joanna Briggs Institute checklists [24]. Discrepancies in scoring were discussed between the reviewing authors.

### Analyses

Quantitative studies: We used descriptive statistics to summarise information about the study characteristics, samples, and methods. Ninety-five percent confidence intervals were reported for all measures of prevalence and risk ratios where available. Meta-analysis was not conducted due to study heterogeneity.

Qualitative analysis: We conducted a thematic analysis of selected articles and used NVIVO 12 to organise the data. All articles were read multiple times by two reviewers (AP, JM) to identify initial codes related to the associations between family violence and mental health and the influence of gender relations (Additional file 1: Appendix), drawing on the accounts of both research participants and the article’s authors. AP and JM discussed these codes to arrive at a consensus and collaboratively developed a final codebook. One reviewer (JM) then went through each article in detail, systematically extracting all relevant quotes. As a descriptive account of the current literature on this topic, further theoretical interpretation beyond a descriptive analysis was not needed.[25].

## Results

### Studies included

**Fig. 1:**
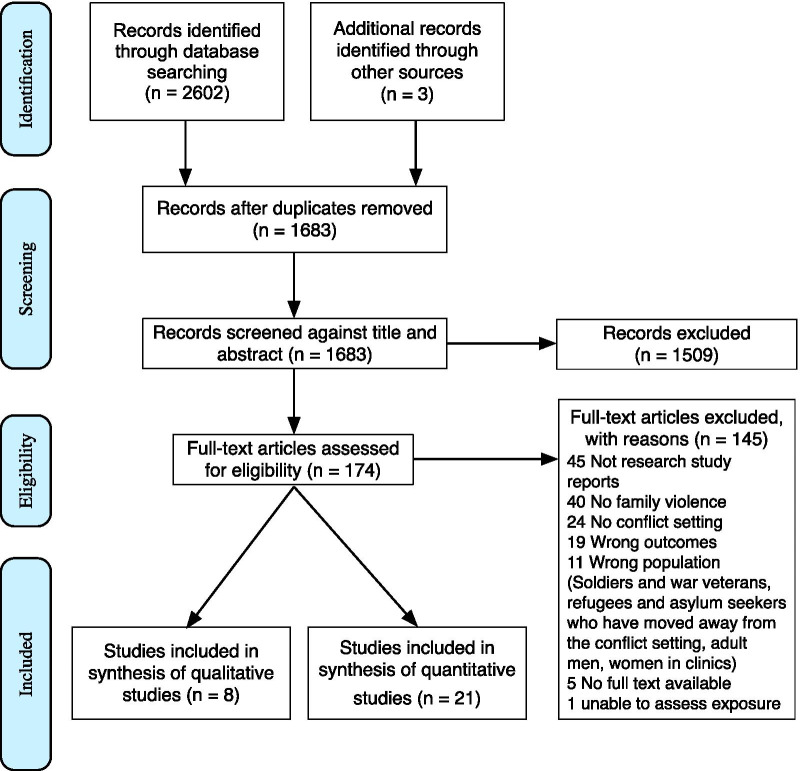
PRISMA flow chart

We identified 2603 records, of which 1682 were duplicates (Fig. 1). Of the 173 full-text articles retrieved, 29 studies conducted between 1994 and 2017 in 13 countries were included (Additional file 1: Appendix). Reasons for exclusion were mostly due to the study not including family violence or not being a full research paper (Additional file 1: Appendix).

The quantitative papers described violence against women and violence against children. We included 14 quantitative studies of violence against women in conflict from 11 countries (five in Africa, five in Asia, one in Europe). All were cross-sectional, with the exception of one cohort study [26]. The sample sizes for women within these studies ranged from 80 to 2196 individuals (Table 1). The types of violence included varied. Eleven studies focused on intimate partner violence (IPV). The other studies had broader definitions including violence from other members of the family and other forms of violence (Table 1).

**Table 1:** Study characteristics: Violence against women

Author	Country	Study setting (urban/rural)	Nature of conflict	Study design	Sample size (n)	Age of sample	Particular group of focus	Type of Violence and Role of perpetrator
Avdibegovic (2006) [37]	Bosnia and Herzegovina	Urban and Rural	Internationalised intrastate	Cross-sectional study	283	16 + ;43 (9.6)	Women in the general population*	Physical, emotional/ psychological and sexual (domestic violence, sexual abuse, psychological abuse) from husbands
Gupta (2014) [16]	Côte d'Ivoire	Rural	Intrastate	Cross-sectional study	(n) = 950	18 + ; 37.4 (11.4)	Those reported to have a male partner at the time of the survey	IPV, domestic violence and rape from partner
Heath (2012) [26]	Palestine	Rural and urban	Intrastate	Cohort study	N = 746 (n) = 383	18–78; 34.68 (12.19)	Living in the West bank, Gaza strip and East Jerusalem	Domestic violence
Hossain (2014) [39]	Côte d'Ivoire	Rural	Intrastate	Cross-sectional study	N = 2678 (n) = 1411	15–49	Resident in the community for one year and access to the International Rescue Committee (a humanitarian organisation)	Domestic violence, marital rape from partner or family member
Jewkes (2018) [13]	Afghanistan	Rural	Internationalised interstate	Cross-sectional study	(n) = 1463	14–48; 29.28	Women who were interested in participation for interventions	IPV from spouse
Johnson (2010) [44]	Democratic Republic of Congo	Rural	Intrastate	Cross-sectional study	N = 998 (n) = 593	18 +	Households in the Eastern DRC	Physical and sexual violence from spouse or partner
Kane (2018) [51]	Iraq	Unknown	Internationalised intrastate	Cross-sectional study	N = 894 (n) = 457	35.69 (14.10)	Adults reporting or witnessing one of eight possible traumatic events	Physical violence from partner
Kinyanda (2013) [49]	Uganda	Unknown	Interstate	Cross-sectional study	N = 1568 (n) = 903	15 +	Non-vulnerable and vulnerable individuals were selected**	IPV from partner or spouse
Kinyanda (2016) [48]	Uganda	Unknown	Interstate	Cross-sectional study	N = 1110 (n) = 694	14 +	Resident of the 4 sub-counties, within the age range of 14 + , conversant with the Itesot language and would understand the survey	Physical, emotional/psychological and sexual violence from partner or spouse
Rees (2016) [45]	Timor-Leste	Facility-based	Intrastate	Cross-sectional study	(n) = 1672	< 15 to > 35	Pregnant women in second trimester	IPV from partner
Shuman (2016) [30]	Côte d'Ivoire	Urban	Intrastate	Cross-sectional study	(n) = 80	18 +	Women from the general population	IPV from partner
Sriskandarajah (2015) [41]	Sri Lanka	Unknown	Intrastate	Cross-sectional study	N = 569 (n) = 122	37.6 (5.6)	Parents of primary school children	Physical, emotional/psychological, sexual violence and IPV from husband
Usta (2008) [50]	Lebanon	Facility-based	Intrastate	Cross-sectional study	(n) = 310	15–72; 36.20 (10.60)	Women from the general population	IPV and domestic violence from husband or family members
Vinck (2013) [38]	Liberia	Rural and urban	Intrastate	Cross-sectional study	N = 4501 (n) = 2196	35.4	Liberian adults	IPV from partner or spouse

^*^The study included women receiving psychiatric treatment. These women were excluded from our analysis

^**^ Vulnerable individuals defined as 'A person who had any of the following characteristics: widowed, divorced, or separated; living in an internally displaced persons camp; women who had suffered sexual torture; single mothers; orphans; out of school youth; child/adolescent mothers; women and adolescent girls without any source of livelihood (mainly lack of access to arable land); having a mental health problem; survivor of intimate partner violence; and survivors of recent famines or floods

We included eight studies of violence against children in conflict-affected countries. All were from Asia except one from Uganda. All were school-based samples except two that were community-based [27, 28], which were the smallest (n = 149 [27]) and largest (n = 513 [28]). Six studies were cross-sectional and two were cohorts (Table 2).

**Table 2:** Study characteristics Violence against children

Author	Country	Study setting (urban/rural)	Nature of conflict	Research design	Sample size (n)	Age of sample	Particular group of focus	Type of violence and role of perpetrator
Catani (2008) [12]	Sri Lanka	School-based	Intrastate	Cross-sectional study	296	9–15; 12.2	Students	Physical abuse, sexual abuse and emotional abuse including witnessing IPV between parents, from family member
Catani (2009) [40]	Afghanistan	School-based, urban	Internationalised intrastate	Cross-sectional study	287	7–15; Girls 11.8 (1.6) Boys 10.9 (1.7). Combined mean: 11	Students	Physical violence (family violence defined as being exposed to physical, emotional or sexual abuse or witnessing IPV) from family member including sibling
Fayyad (2017) [47]	Lebanon	School-based, peri-urban	Intrastate	Cross-sectional study	252	14.7 (1.7)	Adolescents from grades 7 to 12 who experienced at least one war event	Physical violence (family violence) from parents and family
O'Leary (2018) [27]	Afghanistan	Community-based, rural and urban	Internationalised intrastate	Cross-sectional study	149	12–18; 14.6	Children living in Kabul	Physical abuse (domestic violence and neglect) from parents
Panter-Brick (2011) [43]	Afghanistan	School-based	Internationalised intrastate	Cohort study	234	11–16; 13.5 (1.51)	Students	Physical violence (domestic violence) from family members
Panter-Brick (2015) [42]	Afghanistan	School-based	Internationalised intrastate	Cohort study	331	11–16	Students in grades 5–10	Physical violence (domestic violence) from unknown groups
Saile (2016) [28]	Uganda	Community-based	Interstate	Cross-sectional study	513	6–13; 8.79 (SD 1.29)	Children attending second-grade	Physical (family violence e.g. experiencing and witnessing physical and verbal domestic violence) from family member
Sriskandarajah (2015) [41]	Sri Lanka	School-based	Intrastate	Cross-sectional study	359	7–11. M = 9.2 (SD 1.0)	Primary school children and their parents. Tamil families	Physical, emotional/psychological and sexual violence (Exposure to family violence and IPV) from Parents

Eight qualitative studies were included [29–36], presenting research from six countries: Côte d’Ivoire (2), Timor-Leste (2), Colombia (1), Uganda (1), Democratic Republic of the Congo (1), and Sri Lanka (1). Five reports focused exclusively on family violence perpetrated against women, two on violence by mothers against their children, and one on both forms of family violence. Only one report discussed VAW by family members other than the husband. Seven reports used interviews or focus groups and one used a questionnaire. Terms such as depression, anxiety, post-traumatic stress disorder, or suicidal ideation were rarely used. As such, mental health problems were coded as anything that discussed a psychological symptom or negative feelings, including stress, fear, worry, unhappiness, or concern.

We summarise below the results according to our four objectives, including the qualitative and quantitative results together.

1. Prevalence of family violence in conflict-affected areas

The prevalence of family violence and associations with mental health are shown in Tables 3 (for women) and 4 (for children). Amongst women, the prevalence of violence varied, but exposure to domestic violence in both conflict and post-conflict settings was common. The highest prevalence was a study from Bosnia and Herzegovina, where 78.9% of women reported physical abuse and 96.1% psychological abuse [37]. Commonly, however, reports of IPV were in the region of 30–40%. Lifetime experience of IPV was reported at 37.7%[38] from Liberia, 44.6% for those who had 2–4 trauma exposures in a study from Afghanistan [13], and 49.8%in a study from rural Côte d’Ivoire [39]. High prevalence was also reported for exposure to IPV within the last 12 months (24.4% [38], 25.1% [13] and 29.7% [39]). No obvious difference was seen by country or region of the world.

**Table 3:** Violence against women results

Author	Prevalence of family violence	Prevalence of a mental health problem	Evidence of association between family violence and mental health problem
Avdibegovic (2006) [37] Bosnia and Herzegovina	78.9% experienced physical abuse, 60.5% experienced sexual abuse and 96.1% experienced psychological abuse	General neuroticism measured by Cornell Index test was high in 26 and moderate in 30 out of 76 survivors of domestic violence	76% of participants experiencing domestic abuse had symptoms of neurosis General levels of neurosis = 43% (IQR 25.3, 62.3), Anxiety = 64% (IQR 36.0, 73.0), Phobia = 57% (IQR 29.0, 86.0), Depression = 50% (IQR 0.0, 86.0), Obsessive–compulsive tendencies = 50% (IQR 17.0, 67.0)
Gupta (2014) [16] Côte d’Ivoire	Women experiencing lifetime IPV: 26.5% Past year IPV: 23.4%	Of the women who experienced no IPV, 9.2% had probable PTSD Of the women who experienced IPV within the lifetime, but not within the past year 12.3% had probable PTSD 22.1% of the women who experienced past year had probable PTSD	Adjusted OR, for lifetime IPV prior to the past year and PTSD = 1.6 (95% CI 0.9, 2.6) For past year IPV and PTSD = 3.1 (95% CI 1.8, 5.3)
Heath (2012) [26] Palestine	At 18 month follow up: 23.8% reported being insulted, 20.4% pushed/shoved, 14.4% threatened, and 18.3% hit		
Hossain (2014) [39] Côte d’Ivoire	IPV among ever-partnered: 29.1% reported life-time sexual violence, 14.9% in the last 12 months 38.4% reported life-time physical violence, 20.9% in the last 12 months 23.9% reported severe life-time physical violence. 11.6% in the last 12 months 49.8% reported life-time physical and/or sexual violence. 29.7% in the last 12 months		
Jewkes (2018) [13] Afghanistan	Past 12 months physical IPV: Of the women who had no exposure to trauma, 17.7% reported IPV, of those who had 1 trauma exposure; 27.6% reported IPV, and for those who had 2–4 trauma exposures, 25.1% reported IPV (p = 0.006) Lifetime physical risk IPV: Of the women who had no exposure to trauma; 28.1% reported lifetime IPV, among women who had 1 trauma exposure, 47.0% reported lifetime IPV, and for those who had 2–4 trauma exposures, 44.6% reported lifetime IPV (p < 0.0001)		Depression: Of the women who had no exposure to trauma; 12.05% reported depression. Of the women with 1 trauma exposure 16.25% reported depression, and of the women who had 2–4 trauma exposures, 17.1% reported depression (p < 0.0001) PTSD: Of the women who had no exposure to trauma 1.35% had PTSD. Of the women with 1 trauma exposure 1.7% had PTSD. Of the women who had 2–4 trauma exposures 1.65% had PTSD (p < 0.0001)
Johnson (2010) [44] Democratic Republic of Congo	30.5% reported IPV. Of this group, 96% reported physical IPV and 8.5% reported sexual IPV	21.4% reported substance abuse. 41.9% had MDD, 54.0% had PTSD, 27.3% had suicidal ideation and 16.8% reported a suicide attempt	20.5% (95% CI 12.0, 29.0) of women affected by IPV suffered from substance abuse, 64.9% (95% CI 52.3, 77.5) suffered from MDD, 77.2% (95% CI 66.8, 87.7) suffered from PTSD, 42.4% (95% CI 28.9, 56.0) suffered from suicidal ideation, and 33.1% (95% CI 22.9, 43.4) reported a suicide attempt
Kane (2018) [51] Iraq	36.3% domestic violence, 2.4% sexual assault	Depression: HPS-25 M = 1.54 (SD 0.54) p < 0.0001, Post-traumatic stress: HTS M = 1.24 (SD 0.50) p < 0.0001	Linear Regression Beta Coefficient for domestic violence and depression 0.30 (p < 0.0001); and anxiety (p < 0.05) No association with post-traumatic stress
Kinyanda (a) (2013) [49] Uganda	34.5% reported exposure to IPV	14.7% reported problem drinking, 47.9% reported depressive disorder	
Kinyanda (2016) [48] Uganda	44.9% reported any form of IPV 7.8% reported sexual IPV 22.9% reported physical IPV 44.2% reported psychological IPV		Adjusted OR for probable major depressive disorder: physical IPV 1.41 (95% CI 0.96, 2.07), psychological IPV 0.97 (95% CI 0.68, 1.39), sexual IPV 2.15 (95% CI 1.09, 4.23)
Rees (2016) [45] Timor-Leste	30.6% reported severe psychological abuse (threatening, intimidation and controlling) 6.2% reported physical abuse only 19.5% reported combined severe psychological and physical abuse	19.7% met the EPDS threshold for depressive symptoms, 5.7% for PTSD symptoms and 6.3% for psychological distress	Adjusted OR for IPV and Depression EPDS: Severe psychological abuse 1.61 (95% CI 1.17, 2.23). Physical abuse 2.27 (95% CI 1.37, 3.77). Severe psychological and physical abuse 4.3 (95% CI 3.12, 5.96) Adjusted OR for IPV and PTSD: Severe psychological abuse 1.26 (95% CI 0.71, 2.24). Physical abuse 2.20 (95% CI 0.96, 5.04). Severe psychological and physical abuse 3.24 (95% CI 1.90, 5.50) Adjusted OR for IPV and Psychological Distress (Kessler-10 indice): Severe psychological abuse 1.09 (95% CI 0.59, 2.03). Physical abuse 2.08 (95% CI 0.87, 5.01). Severe psychological and physical abuse 5.32 (95% CI 3.20, 8.87)
Shuman (2016) [30] Cote d’Ivoire	53.6% reported exposure to physical, sexual or emotional IPV in the past year. Of the types of violence, 46.4% reported emotional IPV, 21.7% reported sexual IPV and 17.4% reported physical IPV. 29% reported physical and/or sexual IPV in past year Among partnered women only 14.5% reported being pushed, shoved, kicked or dragged. 13% reported being slapped, having something thrown at them or being hit with something. 15.9% reported being forced to have sex as result of threats or intimidation, 11.6% reported being physically forced to have sex, 36.2% reported being frightened or humiliated	10.9% faced mental health repercussions (undefined)	
Sriskandarajah (2015) [41] Sri Lanka	70.3% reported IPV in the past year, 3.4 (SD 4.2) IPV-related events in the past year	27.9% of mothers met criteria for a diagnosis of PTSD	
Usta (2008) [50] Lebanon	27% reported at least one incident of domestic abuse during conflict. 13% reported at least one incidence perpetrated by a husband or other family member		Correlation between negative mental health score and domestic violence during conflict: 0.21 p < 0.001 Correlation between negative mental health score and domestic violence after conflict: 0.14 p < 0.01
Vinck (2013) [38] Liberia	Among adult women, 37.7% (95% CI, 34.9, 40.5) reported lifetime exposure to intimate-partner physical violence. 24.4% (95% CI 22.1, 26.9) reported incidence of intimate-partner physical violence over a one-year recall period Women were 3.3 times more likely than men to report having experienced a severe beating by a spouse or partner	18.8% reported PTSD symptoms 17.6% reported depression symptoms	

CI, Confidence Interval; CMD, Common Mental Health Disorders; CTS, Conflict Tactics Score; EPDS, Edinburgh Depression Scale, GAD, Generalised Anxiety Disorder; HPS-25, Hopkins Symptom Checklist-25; HTS, Harvard Trauma Scale; IQR, Interquartile range; MDD, Major Depressive Disorder; OR, Odds ratio; SD, Standard Deviation; SRQ-20, Self-Reporting Questionnaire-20

**Table 4:** Violence against children results

Author	Prevalence of family violence	Prevalence of a mental health problem	Evidence of association between family violence and mental health problem
Catani (2008) [12] Sri Lanka	95.6% reported at least one family violence event type ever. 64.2% in the last month Children experienced or witnessed 5.3 (SD = 3.2) violent event types. 68.8% of children reported being beaten with an object. 18% of children had suffered at least one injury because of the violent treatment, and 10% of them needed medical treatment. 55.4% reported having witnessed other family members being hit 4.3% reported having experienced or witnessed at least one incident of sexual abuse or sexual violence at home	PTSD: 30.4% (28.5% boys; 32.6% girls); Major Depressive Disorder: 19.6%; Past suicidality: 22.6%; Current suicidal ideation: 17.2%	Exposure to war predicted family violence (p < 0.001) Exposure to family violence predicted PTSD symptoms (p < 0.001)
Catani (2009) [40] Afghanistan	77% (71.3% girls, 81.3% boys) reported at least one type of family violence event ever. 35.2% in the last month Children witnessed on average 4.3 violent event types within family (mean boys = 5.0, girls: 3.5) 23.2% had ever witnessed other family members being hit. 31.5% had witnessed their mother being beaten by the father 41.6% children reported being beaten by their father. 59.9% children reported being beaten by mother. 11% children suffered at least one injury (bruises, bleeding and broken bones)	14.1% and 26.1% of the boys fulfilled all DSM- IV criteria for probable PTSD	Exposure to war predicted family violence in girls (p < 0.001) but not boys Correlation between family violence and PTSD (p < 0.01)
Fayyad (2017) [47] Lebanon	Prevalence of violence in the overall group not given	38% of the students had post-war mental health problems as measured by the SDQ plus impact variable 36% had a CRIES score above 30 (PTSD) Degree of war exposure was a significant predictor of both SDQ (OR 1.42; 95% CI 1.12–1.80) and CRIES (OR 1.41; 95% CI 1.11–1.80)	Stratified by SDQ score (Low SDQ score group = no problem; High or borderline SDQ score = postwar problem) Parents hit: Low SDQ score group = 2% parents hit; high SDQ score = 13.7% (p = < 0.001) Parents hit each other: Low SDQ score group = 2% parents hit; high SDQ score = 11.6% (p = 0.001) Faced family violence: Low SDQ score group = 17.5% parents hit; high SDQ score = 37.4% (p = 0.001) Stratified by CRIES score (CRIES < 30 = not indicating PTSD; ≥ 30 indicating PTSD) Family violence: CRIES < 30 = 19.8%; CRIES ≥ 30 = 35.2% (p value = 0.013)
O'Leary (2018) [27] Afghanistan	71% of reported some level of physical violence At home: 36.2% were hit or hurt, 86.1% screamed at aggressively, 70% called names, 70.7% were pushed, grabbed or kicked; no child reported sexual abuse		
Panter-Brick (2011) [43] Afghanistan	Family violence was reported in 47% by at least one informant over the previous year 11.5% family-level violence (severe physical beatings)		Family violence (severe beating) was associated with an increase in SDQ (p = < 0.01) and CRIES (p = < 0.05) scores but not depression (DSRS) For SDQ, scores increased by 1.85 points (CI 0.03, 3.66) with traumatic beatings and 1.26 points (CI 0.50, 2.03) with a family member who is violent at home
Panter-Brick (2015) [42] Afghanistan	27% report domestic violence in the last year	Mental health was measured at two timepoints T1 and T2. Mean scores at each time point were: CRIES: T1 = 8.0; T2 = 5.9 DSRS: T1 = 9.5; T2 = 7.3 SDQ:T1 = 9.0; T2 = 9.4	Adjusted OR: 4.84 (p < 0.01) stressor of domestic violence causing sustained distress
Saile (2016) [28] Uganda	88.9% were exposed to at least one event from the family violence spectrum. Mean number of family violence adverse events = 3.85 (SD = 3.33) 77% experienced being hit with an object 53% experienced acts of verbal abuse 42% were threatened 38% reported to have witnessed other family members being beaten, punched, or kicked Severe physical maltreatment: 15% were punched or kicked on the body 3% were burnt with hot water or a cigarette on purpose 4% of children were threatened to be killed	PTSD = 3.3–7.2% Depression = 26.3–38.2%	Adjusted model for the association between family violence and mental health outcomes: SDQ = beta coefficient 0.24 (p < 0.001) CDI score (depression symptoms) = beta coefficient 0.17 (p < 0.001) UPID score (PTSD) = beta coefficient 0.11 (p < 0.05) Mediation pathways Model assessing the effect size of traumatic exposure leading to family violence to child psychopathology: SDQ = 0.17 (95% CI 0.10, 0.27) CDI score (depression symptoms) = 0.07 (95% CI 0.02, 0.12) UPID score (PTSD) = 0.06 (95% CI -0.01, 0.15) Model assessing the effect size of traumatic exposure leading to family violence to child psychopathology, mediated by perceived maternal care: SDQ = 0.07 (95% CI 0.03, 0.11) CDI score (depression symptoms) = 0.04 (95% CI 0.02, 0.07) UPID score (PTSD) = 0.04 (95% CI 0.01, 0.08)
Sriskandarajah (2015) [41] Sri Lanka	83.8% reported at least one event of victimisation at home and 71.6% that the violence was in the last month, 76.9% were slapped on body, arms or legs, 44.8% hit with hard objects, 37.3% threatened verbally, 12.8% had at least one injury, 5% needed medical treatment Mean number of intimate partner violence events for the mother is 3.4 in the past year Exposure to mass trauma events was associated with victimisation of the child by both the mother and the father		

CDI, Child Depression Inventory; CI, confidence interval; CRIES, Child Revised Impact of Events Scale. Score of 30 or more indicates PTSD; DSRS, Depression Self Rating Scale; OR, Odds ratio; SDQ, Strengths and Difficulties Questionnaire; UPID, UPID, University of California at Los Angeles PTSD Reaction Index for DSM-IV

The prevalence of violence against children varied but in all cases violence was very common. Lifetime experience of violence was reported at 77% from Afghanistan [40], 88.9% from Uganda [28], and 83.8% and 95.6% in studies from Sri Lanka[12, 41] Similar high prevalences were reported for recent or ongoing violence over the last year (27% [42], 47% [43] and 71% [27]) and last month (35.2% [40], 64.2% [12] and 71.6% [41]). Where the mean number of events was measured, children had experienced 5.3 [12] or witnessed 4.3 [40] family violence events.

2. Risk of mental health problems among women and children who have experienced or witnessed family violence

Nearly all the studies found associations between family violence and adverse mental health among women (Table 3). Depression and PTSD were commonly measured outcomes.

Six studies estimated the association between family violence and mental health outcomes in children. Across all studies, associations were found between family violence and mental ill-health.

### Post-traumatic stress disorder

Gupta et al. [16] found a non-significant positive association between IPV and PTSD (OR 1.6 (95% CI 0.9, 2.6) in Côte d’Ivoire. Johnson et al. [44] found that in the DRC 43.9% (95% CI 34.7, 53.0) of women not affected by IPV had PTSD, compared with 77.2% (95% CI 66.8, 87.7) who were affected by IPV. A dose–response relationship between compound exposures of violence and likelihood of PTSD was reported by Rees et al. [45], whereby severe combined physical and psychological abuse was associated with the greatest risk of PTSD (OR 3.24, 95% CI 1.90, 5.50). These findings were comparable with those found in Afghanistan. Jewkes et al. [13] reported that, of women who had no exposure to trauma, 1.35% had PTSD. Of women with one trauma exposure 1.7% had PTSD, and with 2–4 trauma exposures 1.65% had PTSD (p < 0.0001). Umubyeyi et al.[46] noted strong positive associations between different forms of violence exposure and PTSD in Rwanda: physical (aOR 3.16; 95% CI 1.67, 5.95), sexual (aOR 4.20; 95% CI 2.22, 7.95), psychological (aOR 2.97; 95% CI 1.62–5.45).

In children, studies from Sri Lanka [12], Afghanistan [40, 42, 43], Lebanon [47] and Uganda [28] all showed increases in PTSD associated with family violence. In comparable studies, Catani et al. [12] and Catani et al. [40] showed that exposure to war predicted family violence and PTSD symptoms, and a correlation between family violence and PTSD. Similarly, Fayyad et al. found higher PTSD scores (as measured by the Child Revised Impact of Events Scale (CRIES)) in children who had experienced or witnessed family violence [47].

### Other mental health problems

Umubyeyi et al. [46] found associations between major depressive episode and physical (aOR 4.63; 95% CI 2.57, 8.32), sexual (aOR 5.49; 95% CI 2.94, 10.25), and psychological violence (aOR 5.59; 95% CI 3.19, 9.80). As was the case with PTSD, a dose–response relationship between compound exposures of violence and likelihood of depression was observed. In Afghanistan, Jewkes et al. [13] found that, of women who had no exposure to trauma, 12.1% reported depression; with one trauma exposure, 16.2% reported depression; and of women who had 2–4 trauma exposures; 17.1% reported depression (p < 0.0001). In Bosnia and Herzegovina, Avdibegovic et al. [37] found that 76% ‘domicile’ (community-based sample) participants experiencing domestic abuse had symptoms of neurosis according to the Cornell Index. 17.1% of women who had 2–4 trauma exposures reported depression compared with 12.0% of women who had no exposure to trauma in Afghanistan.

In children, the association with depression was less consistent, though often not measured. Strengths and Difficulties Questionnaire (SDQ) scores, which measure a range of psychosocial outcomes, were also higher in the studies from Lebanon [47] and Afghanistan [42]. Panter-Brick found increases in SDQ score with traumatic beatings and family violence at home [43], and those who had sustained distress compared to the low distress group (measured by CRIES) had an increased risk (adjusted OR 4.84) of living in a family with ongoing stressful domestic violence [42]. Saile et al. examined the pathway between conflict, family violence and mental health in children. They found small changes in SDQ scores, depression symptoms and PTSD [28].

Women’s perspectives on armed conflict or how their experiences of conflict may have affected their mental health were reported in relation to financial stress and their concerns about the care of their children. For instance, women participating in focus group discussions in Côte d’Ivoire reported that financial stress made them lose interest in having sex with their husbands, to which some husbands might respond with sexual violence:

When the woman is not at ease… you know, there is no money to properly take care of the children, she is preoccupied and she does not feel like having sex so it happens that the man rapes her [36].

Another study of returned female combatants in Northern Uganda explored the stresses women felt about the care of their children when they returned from situations in which they had been abducted and forced to marry combatants:

When I had just returned from the bush with my children, I used to have lots of thoughts [worries] on how I will look after the children in case of sickness since their father is not there [34]^.^

Rees et al.[29] uniquely explored the use of violence by women as a result of the armed conflict in Timor-Leste, with the concept of ‘explosive anger’ as an explanation for high rates of violence against children.

3. Variation of the association of family violence with mental health by type of conflict, sex of perpetrator, type of violence

### Type of conflict

In the quantitative studies, we were unable to directly answer our question as to whether the association between family violence and changes in mental health differed by type of conflict. Only the three studies from Uganda were interstate [28, 48, 49]. No discernable difference could be seen between these and the intrastate conflicts (Democratic Republic of Congo [44], Côte d’Ivoire [16, 30, 39], Lebanon [47, 50], Liberia [38], Palestine [26] and Sri Lanka [12, 41]), or the internationalised intrastate conflicts (Afghanistan [13, 27, 40, 43], Bosnia and Herzegovina [37] and Iraq [51]).

The qualitative studies also did not engage with this concept and there were no comparative case studies in the review.

### Sex of perpetrator

Limited data were available on the prevalence of female-perpetrated family violence, and we were unable to assess whether the association between family violence and mental health problems varied according to the sex of the perpetrator. The majority of studies measuring violence against women focused on male spouses in the context of heterosexual married relationships. Two studies, Usta et al. [50] and Hossain et al. [39], defined domestic violence as including violence from family members. In rural Côte d’Ivoire, Hossain et al. [39] demonstrated the lifetime prevalence of sexual violence from female perpetrators including female family members was 0.1%. Among women, lifetime prevalence of physical violence from female family members was 8.9%; prevalence of lifetime physical violence from male family members was also 8.9%.

### Type of violence

We were unable to assess whether the association between family violence and mental health problems varied according to type of violence; only one study provided suitably disaggregated data. Umubyeyi et al. [46] found that PTSD and generalised anxiety disorder (GAD) were most strongly associated with sexual violence (PTSD aOR 4.20 (95% CI 2.22, 7.95); GAD aOR 6.37 (95% CI 3.45,11.79)), followed by physical (PTSD aOR 4.70 (95% CI 2.65, 8.35); GAD aOR 3.16 (95% CI 1.67,5.95)) and psychological violence (PTSD aOR 4.34 (95% CI 2.54, 7.43); GAD aOR 2.97 (95% CI 1.62, 5.45)). Physical violence was more closely associated with suicide risk followed by psychological violence and sexual violence. Major depressive episode showed the strongest association with psychological violence, followed by sexual violence, and physical violence.

4. Impact of gender norms on the mental health of women and children experiencing family violence

The conflict-related triggers identified in qualitative articles, such as financial stress and alcohol use, were explained by both authors and study participants as rooted in gender norms. In situations of conflict, men may not be able to fulfill their socially-expected roles such as providing financially for the household [35], and in some cases women become the main household providers [30]. This has indirect impacts on the mental health of women and children by increasing men’s stress and sense of insecurity in ways that increase family violence [29, 30, 35].

Guruge et al. explained how the use of violence by men becomes a means of maintaining a sense of power and control in the face of the instability brought on by conflict, which threatened gender norms that men rely upon for their identity and sense of security:

With death, disappearances, disability and loss of traditional sources of livelihood (such as fishing) affecting many men, women in war-affected areas of Sri Lanka have taken on being the breadwinner and head of household roles, and have become more active in their communities. When there is a disruption of established social relationships and roles in war and post-war contexts, men are known to use violence as means of re-exerting control and power to maintain roles that are consistent with social norms. [35]

Related to this, economic instability often opens up new opportunities for women to earn, which men may feel threatened by, contributing to their use of violence [29, 30, 35]. Shuman et al., for example, discussed how change in women’s roles arose in Côte d’Ivoire as an underlying cause of family violence:

In post-crisis Côte d’Ivoire, economic opportunities are scarce and in some cases, women have become the financial providers for their families. While women welcomed opportunities to have more control over resources in their relationships, they also described being perceived as a threat by their partners. Among men, this perceived loss of control and traditional gender responsibilities was discussed as an underlying cause of all forms of IPV. [30]

The stress reported by men was also highlighted as a reason for their increased drinking behaviour. In Côte d’Ivoire, Northern Uganda and Sri Lanka, instability and financial problems arising from the conflict were seen as triggers for problematic drinking by men, which contributed in turn to increased episodes of violence[34–36].

The qualitative data also support the impact of gender norms on the mental health of women and children experiencing violence directly through their experience of violence-related stigma. [29, 30]. Sexual violence was frequently described as especially stigmatising for women. For instance, Annan and Brier’s study of women abducted by the Lord’s Resistance Army in Northern Uganda described the lower status of women who return to their communities as women who had been ‘married’ or sexually abused. They described how the fear of this stigma drives women to agree to be married either to violent men or as second wives, which is attached to lower status in the household, in turn increasing the risk of violence:

While polygamous marriages are common, women as second wives are seen to have less power in households than first wives. One social worker observed that women’s insecurities about social status combined with economic pressures to push them into relationships faster than their peers – some entering negative relationships, confirming their insecurities about having less value than other women. [29]

Annan and Brier (2010) also argued that forced marriage as part of armed conflict may have similar long-term psychological impacts on women as childhood experiences of violence, with similar consequences for difficult relationships with men in adulthood.

The qualitative results predominantly related to violence against women, but one study, Kohli et al. (2015), highlighted how the stigmatisation of violence against women has affected the mental health of children by contributing to social isolation in South Kivu, DRC:

Further, participants described how living in an unstable and violent household negatively affected children’s interaction with community members, as the entire family could become isolated and disrespected in the community. Male and female participants in this study described households with IPV as unable to progress or have stability in their lives. [31]

## Study quality

Quality assessments for both quantitative and qualitative studies are in the Additional file 1: Appendix. Overall, there was a low risk of bias in most of the quantitative studies. Bias existed in identifying and dealing with confounding, which we assessed as high or unclear in seven studies [30, 37, 40, 45, 48, 50]. Sampling criteria were defined in all studies, with most adopting a version of random sampling. In some, for example O’Leary et al. [27], purposive sampling was used, leading to a likely bias.

The qualitative studies were generally of high quality, with the exception of the categories of cultural and theoretical positionality and reflexivity, for which we felt that all studies were lacking.

## Discussion

Conflict has a pervasive impact on people and societies. Conflict-related violence is well documented, but less well known is how exposure to armed conflict and its social and economic consequences can influence family violence and mental health problems. Our results provided evidence that family violence is common and is associated with poor mental health outcomes, but variability in outcomes and measurement meant that we were not able to support or refute the hypothesis that the prevalence increases in conflict settings nor that it changes the association with mental disorders.

Worldwide, approximately one in three women have survived physical or sexual violence, but the prevalence varies greatly by location. By WHO region, lifetime prevalence of physical or sexual intimate partner violence is highest in the Pacific (Melanesia 51%, Micronesia 41%, Polynesia 39%), South Asia (35%) and sub-Saharan Africa (33%) [52]. According to a recent WHO report, many countries with a recent history of armed conflict, including Afghanistan and Papua New Guinea, have some of the highest prevalences of physical and sexual IPV (46% and 51%, respectively), but data from conflict settings remain poor [52]. Our review found similar proportions, but the studies varied widely, most likely representing actual differences in prevalence, but also methodological issues such as study design, data availability, and tools used.

Studies of violence against children showed the almost ubiquitous presence of violence. It is estimated that one billion children have suffered from violence in the preceding year [4]. A systematic review by Hillis et al. from 96 countries (n = 38) summarised the prevalence of violence over the past year in any country. In Africa, Asia and North America, approximately half to two-thirds of children suffered violence. Prevalence in Latin America, Oceania, and Europe was a little lower, mostly at around one-third[4]. Direct comparison is limited as the countries included in the review by Hillis et al. did not overlap with ours, with the exception of Uganda, where they reported data from a randomised controlled trial [53]. Studies in our review reported recent violence at a similar prevalence in the studies from Afghanistan. The two Sri Lankan studies appeared to show a higher prevalence, reporting 64.2% [12] and 71.6% [41] in the past month alone. Amongst Violence Against Children and Youth Surveys (VACS) studies, only one country – Uganda – has comparable data [54]. This study reported a similar prevalence of lifetime family violence (59% against girls and 68% against boys). Conflicts themselves vary and the impact on individual families is different. Other factors may also affect health. For example, in a study from Sri Lanka, the population also experienced the Indian Ocean tsunami [12].

The finding of an association between violence and mental health problems is not new [55, 56], and was reported in approximately half the studies of violence against women in our review. The studies were not amenable to meta-analysis, nor could comparisons be drawn readily with non-conflict settings. The most consistent association was with PTSD. This may be due to an increased risk of PTSD, as has been commonly shown in conflict-affected populations, but may also reflect a bias in the topics that are researched. For studies that included suicide risk and substance use as additional outcome measures, the association between exposure to family violence and prevalence broadly followed the dose–response relationship observed for PTSD.

Amongst studies of violence against children, consistent associations were found between family violence and symptoms of mental health problems. Three studies constructed structural equation models to explore associations [13, 26, 28]. Heath et al. [26] and Jewkes et al. [13] treated violence and mental health problems (PTSD and depression) as co-outcomes and did not examine the association between the two. Jewkes et al. showed the importance of household wealth and previous childhood trauma [13]. Saile et al.[28] proposed maternal care as a mediating factor that could reduce adverse mental health symptoms.

Armed conflict can challenge dominant masculinities that cast men as providers and heads of family in ways that contribute to perpetration of family violence [31, 33, 36]. Gender norms undermine women’s mental health during conflict by contributing to shame and stigma associated with experiencing both sexual violence and family violence more broadly, and this can also contribute to the social isolation of children [31]. However, the same destabilising effects of armed conflict can also give women new economic opportunities and contribute to their economic and social empowerment [29, 30, 35].

### Strengths and limitations

The main strengths of the review included its robust search strategy, its use of dual reviewers, and analysis and synthesis of both quantitative and qualitative data. The review protocol was prospectively registered and the reporting followed established standards. Limitations should, however, be noted. Studies were highly heterogeneous and meta-analysis could not be conducted and pooled estimates of the association between exposure to family violence and mental health problems could not be calculated. Violence is likely to have been under-reported, and the extent of under-reporting and other biases in data collection would vary between studies and settings. Although a standardised approach for research on violence against children has been advocated (the Violence Against Children and Youth Surveys (VACS)), the studies included in the review did not follow this approach. Variation in outcomes would in part be due to the definitions used, with no set definition of family violence being accepted [57]. Our definition of family violence resulted in the inclusion of studies of violence against women and violence against children, but rarely was family violence explicitly defined within the studies.

Although we found consistent evidence of association between exposure to family violence and mental health problems for both adults and children, we can make limited inferences regarding causality due to the predominance of cross-sectional studies within the review. It is possible that both family violence and changes in mental health are outcomes of exposure to conflict, as described by Heath et al. [26] and Jewkes et al. [13], and reverse causality may occur [58].

In reviewing the qualitative studies, two forms of maltreatment of women (and children) not captured by our search terms are potentially salient for women’s mental health and could be rationalised under a broader conceptualisation of family violence. First, rejection and neglect following women and children’s return from conflict-related assault or time embedded with conflict groups affects their ability to reintegrate into family life, with implications for mental health. Second, sexual violence by combatants to entrap girls and women into forced unions could be viewed as initiating acts of both intimate partnerships and IPV.

## Conclusions

Our systematic review showed that family violence was common in conflict settings and was associated with mental health outcomes. However, we were unable to determine whether prevalence or risk was greater than in non-conflict settings. This gap in the review stems from a lack of comparable data and reiterates the need for standardised measurement of both violence against women and violence against children. It also draws attention to the need to break down existing silos between research on violence affecting women and that affecting children to build a more comprehensive picture of the overlapping risks and mental health outcomes of violence within families [59]. A far more robust understanding of the impacts of armed conflict on families and their experiences of violence is urgently needed to develop comprehensive support systems in conflict-affected settings.

## Supplementary Information

**Additional file 1:** PRISMA checklist, search terms, coding framework for qualitative papers and quality assessments.

## Data Availability

Data sharing is not applicable to this article as no datasets were generated or analysed during the current study.
