# Supplementary material for: Mental health of women and children experiencing family violence in conflict settings: a mixed methods systematic review
Source: Confl Health. 2021 Oct 15;15:74. doi: 10.1186/s13031-021-00410-4 (PMC8518246; doi:10.1186/s13031-021-00410-4)
Supplement: Supplementary file 1 — Additional file 1: PRISMA checklist, search terms, coding framework for qualitative papers and quality assessments. [file 13031_2021_410_MOESM1_ESM.docx]

**Appendix**

**Mental health of women and children experiencing family violence in conflict-affected areas: a systematic review of qualitative and quantitative evidence**

**Table of contents**

[PRISMA checklist 1](#_Toc73533297)

[Search terms 3](#_Toc73533298)

[Coding framework for qualitative papers 4](#_Toc73533299)

[Quality assessments 5](#_Toc73533300)

## PRISMA checklist

(from Moher D et al, The PRISMA Group (2009) Preferred Reporting Items for Systematic Reviews and Meta-Analyses: The PRISMA Statement. PLoS Med 6(7): e1000097)

| Section/topic | # | Checklist item | Reported on page # |
| --- | --- | --- | --- |
| **TITLE** | | | |
| Title | 1 | Identify the report as a systematic review, meta-analysis, or both. | Title |
| **ABSTRACT** | | | |
| Structured summary | 2 | Provide a structured summary including, as applicable: background; objectives; data sources; study eligibility criteria, participants, and interventions; study appraisal and synthesis methods; results; limitations; conclusions and implications of key findings; systematic review registration number. | 2 |
| **INTRODUCTION** | | | |
| Rationale | 3 | Describe the rationale for the review in the context of what is already known. | 4 |
| Objectives | 4 | Provide an explicit statement of questions being addressed with reference to participants, interventions, comparisons, outcomes, and study design (PICOS). | 4 and 6 |
| **METHODS** | | | |
| Protocol and registration | 5 | Indicate if a review protocol exists, if and where it can be accessed (e.g., Web address), and, if available, provide registration information including registration number. | 4 |
| Eligibility criteria | 6 | Specify study characteristics (e.g., PICOS, length of follow-up) and report characteristics (e.g., years considered, language, publication status) used as criteria for eligibility, giving rationale. | 4-6 |
| Information sources | 7 | Describe all information sources (e.g., databases with dates of coverage, contact with study authors to identify additional studies) in the search and date last searched. | 4 |
| Search | 8 | Present full electronic search strategy for at least one database, including any limits used, such that it could be repeated. | 5 and appendix |
| Study selection | 9 | State the process for selecting studies (i.e., screening, eligibility, included in systematic review, and, if applicable, included in the meta-analysis). | 7 |
| Data collection process | 10 | Describe method of data extraction from reports (e.g., piloted forms, independently, in duplicate) and any processes for obtaining and confirming data from investigators. | 7 |
| Data items | 11 | List and define all variables for which data were sought (e.g., PICOS, funding sources) and any assumptions and simplifications made. | Tables 1 and 2 |
| Risk of bias in individual studies | 12 | Describe methods used for assessing risk of bias of individual studies (including specification of whether this was done at the study or outcome level), and how this information is to be used in any data synthesis. | 7 |
| Summary measures | 13 | State the principal summary measures (e.g., risk ratio, difference in means). | 6 |
| Synthesis of results | 14 | Describe the methods of handling data and combining results of studies, if done, including measures of consistency (e.g., I^2^) for each meta-analysis. | Not done |
| Risk of bias across studies | 15 | Specify any assessment of risk of bias that may affect the cumulative evidence (e.g., publication bias, selective reporting within studies). | Appendix |
| Additional analyses | 16 | Describe methods of additional analyses (e.g., sensitivity or subgroup analyses, meta-regression), if done, indicating which were pre-specified. | No applicable |
| RESULTS | | | |
| Study selection | 17 | Give numbers of studies screened, assessed for eligibility, and included in the review, with reasons for exclusions at each stage, ideally with a flow diagram. | 8 and Figure 1 |
| Study characteristics | 18 | For each study, present characteristics for which data were extracted (e.g., study size, PICOS, follow-up period) and provide the citations. | Tables 1 and 2 |
| Risk of bias within studies | 19 | Present data on risk of bias of each study and, if available, any outcome-level assessment (see Item 12). | Appendix |
| Results of individual studies | 20 | For all outcomes considered (benefits or harms), present, for each study: (a) simple summary data for each intervention group and (b) effect estimates and confidence intervals, ideally with a forest plot. | Table 1 |
| Synthesis of results | 21 | Present results of each meta-analysis done, including confidence intervals and measures of consistency. | Not done |
| Risk of bias across studies | 22 | Present results of any assessment of risk of bias across studies (see Item 15). | Appendix |
| Additional analysis | 23 | Give results of additional analyses, if done (e.g., sensitivity or subgroup analyses, meta-regression [see Item 16]). | Not done |
| DISCUSSION | | | |
| Summary of evidence | 24 | Summarize the main findings including the strength of evidence for each main outcome; consider their relevance to key groups (e.g., health care providers, users, and policy makers). | 17-19 |
| Limitations | 25 | Discuss limitations at study and outcome level (e.g., risk of bias), and at review level (e.g., incomplete retrieval of identified research, reporting bias). | 20 |
| Conclusions | 26 | Provide a general interpretation of the results in the context of other evidence, and implications for future research. | 21 |
| FUNDING | | | |
| Funding | 27 | Describe sources of funding for the systematic review and other support (e.g., supply of data); role of funders for the systematic review. | 22 |

## Search terms

(((((“Women”[Title/Abstract] OR “Woman"[Title/Abstract] OR "Female"[Title/Abstract] OR "Child"[Mesh] OR "Child*"[Title/Abstract] OR "Adolescent"[Mesh] OR “Teen*”[Title/Abstract] OR "Adolescent"[Title/Abstract] OR "Girl*”[Title/Abstract] OR "Boy”[Title/Abstract] OR "Boys”[Title/Abstract] OR "Female"[Mesh] OR "Women"[Mesh]))) **AND**

(("Mental Health"[Mesh] OR "Mental Disorders"[Mesh] OR "Mental Health"[Title/Abstract] OR "Anxiety Disorders"[Mesh] OR "Panic Disorder"[Mesh] OR "Anxiety, Separation"[Mesh] OR "Mood Disorders"[Mesh] OR "Depressive Disorder"[Mesh] OR "Depressive Disorder, Major"[Mesh] OR "Neurodevelopmental Disorders"[Mesh] OR "Attention Deficit and Disruptive Behavior Disorders"[Mesh] OR "Autism Spectrum Disorder"[Mesh] OR "Substance-Related Disorders"[Mesh] OR "Feeding and Eating Disorders"[Mesh] OR "Personality Disorders"[Mesh] OR "Stress Disorders, Traumatic"[Mesh] OR "Stress Disorders, Post-Traumatic"[Mesh] OR "Psychological Trauma"[Mesh] OR “wellbeing” [Title/Abstract] OR “psychosocial wellbeing” [Title/Abstract] OR “emotional wellbeing” [Title/Abstract] OR “social wellbeing” [Title/Abstract] OR “well-being” [Title/Abstract] OR “psychosocial distress” [Title/Abstract] OR “PTSD” [Title/Abstract] OR "Mental Disorders"[Title/Abstract] OR "Panic Disorder" [Title/Abstract] OR "Anxiety "[Title/Abstract] OR "Mood Disorders" [Title/Abstract] OR "Depressive Disorder"[ Title/Abstract] OR "Neurodevelopmental Disorders"[ Title/Abstract] OR "Attention Deficit and Disruptive Behavior Disorders"[ Title/Abstract] OR "Autism" [Title/Abstract] OR "Substance-Related Disorders"[ Title/Abstract] OR "Personality Disorders"[ Title/Abstract] OR "Traumatic Stress Disorders" [Title/Abstract] OR "Psychological Trauma" [Title/Abstract]))) **AND**

(("Warfare and Armed Conflicts"[Mesh] OR "Armed Conflicts"[Mesh] OR "armed conflict*"[Title/Abstract] OR "War Crimes"[Mesh] OR "War Exposure"[Mesh] OR “political violence”[Title/Abstract] OR “War”[Title/Abstract] OR “Warfare”[Title/Abstract]))) **AND**

(("Intimate Partner Violence"[Mesh] OR “IPV”[Title/Abstract] OR "Domestic Violence"[Mesh] OR "Intimate Partner Violence"[Title/Abstract] OR "Domestic Violence"[Title/Abstract] OR "Child Abuse"[Mesh] OR "Child Abuse"[Title/Abstract] OR "Spouse Abuse"[Mesh] OR "Gender-Based Violence"[Mesh] OR "Gender-Based Violence"[Title/Abstract] OR "Physical Abuse"[Mesh] OR "Rape"[Mesh] OR “physical harm”[Title/Abstract] OR "sexual harm"[Title/Abstract] OR “sexual violence”[Title/Abstract] OR “Family Violence”[Title/Abstract] OR “Child maltreatment”[Title/Abstract] OR ”Battered woman”[Mesh] OR ”Battered woman”[Title/Abstract] OR “Rape”[Title/Abstract]))

## Coding framework for qualitative papers

| **Theme** | **Example quote** |
| --- | --- |
| **Organising theme**: relationship between exposure to armed conflict and mental health | |
| Men’s stress about housing, work, and harmful alcohol use during periods of conflict contributes to their use of violence against women | For housing, how am I going to manage? So I have no income, no savings, how am I going to ﬁnd an activity? All these thoughts make the man anxious and this cannot favour the harmony in the family. (Cardoso et al., 2016, p. 372) |
| Men and women’s stress during periods of armed conflict contribute to their use of violence against their children | Several women attributed their anger and propensity towards harsh parenting to the pressures of caring for many children in conditions of extreme hardship. (Rees et al., 2015). |
| **Organising theme**: Influence of gender norms on mental health and DV in contexts of conflict | |
| Conflict leads to men’s loss of power and control, which magnifies DV | The root cause [of IPV] are [sic] various things related to masculinity and not being able to fulfil your role because of the conflict and not being able to work or contribute in the family. The insecurity and the fear enables that frustration and the anger. (Guruge et al., 2017, p. 8) |
| Women find new sources of income during conflict, destabilising existing power dynamics, magnifying DV | In post-crisis Côte d’Ivoire, economic opportunities are scarce and in some cases, women have become the financial providers for their families. While women welcomed opportunities to have more control over resources in their relationships, they also described being perceived as a threat by their partners. Among men, this perceived loss of control and traditional gender responsibilities was discussed as an underlying cause of all forms of IPV. (Shuman et al., 2016, p. 8) |
| Gender norms stigmatise experiences of sexual violence during conflict | While polygamous marriages are common, women as second wives are seen to have less power in households than ﬁrst wives. One social worker observed that women’s insecurities about social status combined with economic pressures to push them into relationships faster than their peers – some entering negative relationships, conﬁrming their insecurities about having less value than other women. Annan and Brief (2010) argue that forced marriage as part of armed conflict may have similar long-term psychological impacts on women as childhood experience of violence, with similar consequences for difficult relationships with men in adulthood. (Annan & Brier, 2010, p. 156) |
| Displacement magnifies the stigma of DV | The houses are so close to each other and the courtyard is common, so everybody will hear what’s happened in your household. If your husband batters you all the time, everybody will know and you’ll no longer be respected by the neighbors, even their children will disrespect you. That’s why we are so concerned about the others’ ‘eyes.’ (Shuman et al., 2016, p. 7) |
| Insecurity from conflict forces women into marriages, as a form of DV | While polygamous marriages are common, women as second wives are seen to have less power in households than ﬁrst wives. One social worker observed that women’s insecurities about social status combined with economic pressures to push them into relationships faster than their peers – some entering negative relationships, conﬁrming their insecurities about having less value than other women. Annan and Brief (2010) argue that forced marriage as part of armed conflict may have similar long-term psychological impacts on women as childhood experience of violence, with similar consequences for difficult relationships with men in adulthood. (Annan & Brier, 2010, p. 156) |

## Quality assessments

Quantitative studies

| Cross-Sectional Studies | Were the criteria for inclusion in the sample clearly defined? | Were the study subjects and the setting described in detail? | Was the exposure measured in a valid and reliable way? | Were objective, standard criteria used for the measurement of the condition? | Were confounding factors identified? | Were strategies to deal with confounding factors stated? | Were the outcomes measured in a valid and reliable way? | Was appropriate statistical analysis used? |  |  |  |
| --- | --- | --- | --- | --- | --- | --- | --- | --- | --- | --- | --- |
| Avdibegovic (2006) | LOW | LOW | LOW | LOW | HIGH | HIGH | LOW | HIGH |  |  |  |
| Catani (2008) | LOW | LOW | LOW | LOW | LOW | LOW | LOW | LOW |  |  |  |
| Catani (2009) | LOW | LOW | LOW | LOW | HIGH | LOW | LOW | HIGH |  |  |  |
| Fayyad (2017) | LOW | LOW | LOW | LOW | LOW | LOW | LOW | LOW |  |  |  |
| Gupta (2014) | LOW | HIGH | LOW | LOW | LOW | LOW | LOW | LOW |  |  |  |
| Hossain (2014) | LOW | LOW | LOW | LOW | LOW | LOW | LOW | LOW |  |  |  |
| Jewkes (2018) | LOW | LOW | LOW | LOW | LOW | LOW | LOW | LOW |  |  |  |
| Johnson (2010) | LOW | LOW | LOW | LOW | LOW | LOW | LOW | LOW |  |  |  |
| Kane (2018) | LOW | LOW | HIGH | LOW | LOW | LOW | LOW | LOW |  |  |  |
| Kinyanda (2013) | LOW | LOW | LOW | LOW | LOW | LOW | LOW | LOW |  |  |  |
| Kinyanda (2016) | LOW | LOW | LOW | LOW | UNCLEAR | LOW | LOW | LOW |  |  |  |
| O'Leary (2018) | LOW | LOW | LOW | LOW | LOW | LOW | LOW | LOW |  |  |  |
| Rees (2016) | LOW | LOW | LOW | LOW | UNCLEAR | LOW | LOW | LOW |  |  |  |
| Saile (2016) | LOW | LOW | LOW | LOW | LOW | LOW | LOW | LOW |  |  |  |
| Shuman (2016) | LOW | UNCLEAR | LOW | LOW | LOW | UNCLEAR | LOW | LOW |  |  |  |
| Sriskandarajah (2015) | LOW | LOW | LOW | LOW | LOW | HIGH | LOW | HIGH |  |  |  |
| Usta (2008) | LOW | LOW | LOW | HIGH | UNCLEAR | UNCLEAR | LOW | UNCLEAR |  |  |  |
| Vinck (2013) | LOW | LOW | LOW | LOW | LOW | LOW | LOW | LOW |  |  |  |

| Cohort Studies | Were the two groups similar and recruited from the same population? | Were the exposures measured similarly to assign people to both exposed and unexposed groups? | Was the exposure measured in a valid and reliable way? | Were confounding factors identified? | Were strategies to deal with confounding factors stated? | Were the groups/ participants free of the outcome at the start of the study (or at the moment of exposure)? | Were the outcomes measured in a valid and reliable way? | Was the follow up time reported and sufficient to be long enough for outcomes to occur? | Was the follow up complete, and if not, were the reasons to loss to follow up described and explored? | Were strategies to address incomplete follow up utilized? | Was appropriate statistical analysis used? |
| --- | --- | --- | --- | --- | --- | --- | --- | --- | --- | --- | --- |
| Heath (2013) | LOW | LOW | LOW | LOW | LOW | UNCLEAR | LOW | LOW | LOW | LOW | LOW |
| Panter-Brick (2011) | LOW | LOW | LOW | LOW | LOW | HIGH | LOW | LOW | HIGH | LOW | LOW |
| Panter-Brick (2015) | LOW | LOW | LOW | LOW | LOW | HIGH | LOW | LOW | HIGH | HIGH | LOW |

Qualitative studies

| Author | Philosophy and research methodology | Methodology and Research questions or objectives | Methodology and methods | Methodology, representation and analysis | Methodology and interpretation | Cultural theoretical positionality | Reflexivity influence | Voices represented adequately | Clear ethics | Grounded conclusions |
| --- | --- | --- | --- | --- | --- | --- | --- | --- | --- | --- |
| Annan and Brier (2010) | YES | YES | YES | YES | YES | NO | NO | YES | YES | YES |
| Cardoso et al (2016) | YES | YES | YES | YES | YES | NO | NO | YES | YES | YES |
| Guruge et al (2017) | UNCLEAR | YES | YES | YES | YES | NO | NO | YES | YES | YES |
| Hernandez and Romero (2003) | YES | UNCLEAR | YES | YES | NO | NO | NO | YES | NO | NO |
| Kohli et al (2015) | YES | YES | YES | YES | YES | NO | NO | NO | YES | YES |
| Rees et al (2015) | YES | YES | YES | YES | YES | NO | NO | YES | YES | YES |
| Rees et al (2013) | YES | YES | YES | YES | YES | NO | NO | NO | YES | YES |
| Shuman et al (2016) | UNCLEAR | YES | YES | YES | YES | NO | NO | YES | YES | YES |
